# An impossibility theorem in game dynamics

**DOI:** 10.1073/pnas.2305349120

**Published:** 2023-10-05

**Authors:** Jason Milionis, Christos Papadimitriou, Georgios Piliouras, Kelly Spendlove

**Affiliations:** ^a^Department of Computer Science, Columbia University, New York, NY 10027; ^b^Google DeepMind, London EC4A 3TW, United Kingdom; ^c^Google, Mountain View, CA 94043

**Keywords:** game theory, dynamical systems, Nash equilibrium, solution concept

## Abstract

The Nash equilibrium—a, combination of choices by the players of a game from which no self-interested player would deviate—is the predominant solution concept in game theory. Even though every game has a Nash equilibrium, it is not known whether there are deterministic behaviors of the players who play a game repeatedly that are guaranteed to converge to a Nash equilibrium of the game from all starting points. If one assumes that the players’ behavior is a discrete-time or continuous-time rule whereby the current mixed strategy profile is mapped to the next, this question becomes a problem in the theory of dynamical systems. We apply this theory, and in particular Conley index theory, to prove a general impossibility result: There exist games, for which all game dynamics fail to converge to Nash equilibria from all starting points. The games which help prove this impossibility result are degenerate, but we conjecture that the same result holds, under computational complexity assumptions, for nondegenerate games. We also prove a stronger impossibility result for the solution concept of approximate Nash equilibria: For a set of games of positive measure, no game dynamics can converge to the set of approximate Nash equilibria for a sufficiently small yet substantial approximation bound. Our results establish that, although the notions of Nash equilibrium and its computation-inspired approximations are universally applicable in all games, they are fundamentally incomplete as predictors of long-term player behavior.

## Significance

The Nash equilibrium is the fundamental solution concept of game theory, playing a central role in fields beyond economics. Nash’s existence proof is famously nonconstructive, and the dynamics employed in the proof fail to converge to a Nash equilibrium. We show that this failure is a special case of a much more sweeping impossibility result for any game dynamics satisfying a minimal set of desiderata. Thus, the solution concept of Nash equilibria, while universally applicable, is also simultaneously a fundamentally incomplete description of the possible long-term agent behavior.

The Nash equilibrium, defined and shown universal by John F. Nash in 1950 (1), is paramount in game theory, routinely considered as the default solution concept—the “meaning of the game.” Over the years—and especially in the past three decades during which game theory has come under intense computational scrutiny—the Nash equilibrium has been noted to suffer from a number of disadvantages of a computational nature. There are no known efficient algorithms for computing the Nash equilibrium of a game, and in fact, the problem has been shown to be intractable (2–4). In addition, there are typically many Nash equilibria in a game, and the selection problem leads to conceptual complications and further intractability; see, for example, ref. 5.

A common defense of the Nash equilibrium in the face of such shortcomings is the informal argument that “the players will eventually get there.” However, no learning behavior has been shown to converge to equilibrium in all games (6–13). In fact, refs. 8 and 9 show that for wide classes of games, numerous learning dynamics fail to converge to a fixed point of any kind, and instead exhibit chaotic dynamics. The celebrated work of Hart and Mas-Colell (14) shows that even in games with a single Nash equilibrium, learning dynamics which are uncoupled (players only know their own payoff function) fail to converge to the Nash equilibrium. However, the uncoupledness assumption is strong and crucial for this result, and the problem remains open for general dynamics.

Given a game, the deterministic way players move from one mixed strategy profile to the next can be formalized in terms of dynamical systems as a continuous function φ assigning to each point x in the strategy space and each time t≥0 another point φ(t,x): the point where the players will be after time t; naturally, this function must satisfy $\varphi \left(t^{\prime },\varphi \left(t,x\right)\right)=\varphi \left(t+t^{\prime },x\right)$. If t∈R, this setup is called continuous time dynamics, while if t∈Z, it is referred to as discrete time dynamics. For φ to qualify as game dynamics for a game *g*, it must satisfy Nash stationarity: The Nash equilibria are a subset of the fixed points of φ. This is a reasonable assumption, since game dynamics should encode the strategic stability of Nash equilibria, i.e., no rational player will ever move away from a Nash equilibrium.

Having defined what we mean by “game dynamics,” we turn to defining “convergence.” In the theory of dynamical systems, a standard tool for convergence is Lyapunov functions, which provide an intuitive optimization point of view in the analysis of long-term behavior. Informally, these are real-valued functions that are always decreasing except when the dynamics have reached a final set of configurations. The standard example of such dynamics is gradient flows, where the dynamics move in the direction of the steepest descent, and the final configurations are the critical points of the function. The surprising power of Lyapunov arguments lies in explaining the behavior of dynamics that are not gradient-like. Specifically, an arbitrary dynamical system can be described by a decomposition of its domain into the chain recurrent components, on which the dynamics stay within the component, and outside of which the dynamics flows from one component to another (15), guided by a Lyapunov function: a function that is constant along orbits within the chain recurrent components, and strictly decreasing outside of them.

In view of this mathematical framework, we are interested in this question: Are there game dynamics which converge to Nash equilibria for all games and from all starting points? Our main result is an impossibility theorem:

Main Result (informal statement) there are games in which any game dynamics will fail to converge to the set of Nash equilibria.

That is to say, we exhibit games in which any game dynamics must fail to converge to the set of Nash equilibria—or even approximate Nash equilibria—from certain starting points. This suggests that the Nash equilibrium concept is plagued by a form of incompleteness: It is incapable of capturing the long-term behaviors of the players in all games.

This result complements existing literature, in which the nonexistence of Nash-convergent dynamics is shown from stronger assumptions (14, 16). Also note that our impossibility result is complementary to the known intractability of the Nash equilibrium (2–4) and cannot be derived from it. Indeed, it is well known that there are dynamical systems whose next-step function can be computed efficiently and which converge to points of extremely high computational complexity (PSPACE-complete); see, for example, ref. 17; therefore, computational complexity in and by itself does not preclude convergent dynamics.

Two objections can be raised to the impossibility result: Degenerate games (as we use in our example) are known to have measure zero—so are there dynamics that work for almost all games? (As we shall see soon, the answer is “yes, but.”) Second, in view of the known intractability of the Nash equilibrium, exact equilibria may be asking too much; are there dynamics that converge to an arbitrarily good approximation of the Nash equilibrium? We show how our result can be extended so that it addresses these questions.

For the first question, it turns out that, in some sense, degeneracy is required for the impossibility result: We give an algorithm which, given any nondegenerate game, specifies somewhat trivial game dynamics which are Nash convergent. The downside of this positive result is that the algorithm requires exponential time unless P = PPAD—a complexity collapse that is considered unlikely; see, for example, ref. 2. In fact, we conjecture that such intractability is inherent. In other words, we suspect that, in nondegenerate games, it is complexity theory, and not topology, that provides the proof of the impossibility result. Proving this conjecture would require the development of a complexity-theoretic treatment of dynamics, which seems to us a very attractive research direction.

Second, we exhibit a family of games, with nonzero measure (in particular, perturbations of the game used for our main result), for which any game dynamics will fail to converge (in the above sense) to the set of ϵ-approximate Nash equilibria, for some fixed ϵ>0. Note that, because of the nonzero measure claim, this result appears to be stronger than our main result; however, this is not true because it relies on the stronger assumption that all approximate equilibria are stationary.

Our impossibility result also applies to player behaviors with memory, that is, one in which the players maintain and update quantities which affect their choices. If memory is modeled by expanding the space to include memory components, then impossibility can be recovered under assumptions, even though this requires a more detailed treatment. An interesting example is recent work on optimistic dynamics, where agents keep track of the last two periods of play (18–20). Exploring the limits of the applicability of the impossibility result to such dynamics is an intriguing direction for future work.

Finally, we note that our impossibility results do not apply to stochastic dynamics—e.g., discrete-time dynamics in which φ(x) is a distribution of possible next points. In fact, it is easy to see that even trivial such schemes—e.g., “from any point x jump to a uniformly random point in the domain”—succeed in eventually reaching an arbitrarily good approximation of a Nash equilibrium, albeit in exponential time.

## Game Dynamics and Nash Convergence

Before turning to our notion of convergence, we shall discuss what it means for a dynamical system φ to be compatible with a game *g*. Let us denote the Nash equilibria of *g* as NE(g) or simply *NE* when the context is clear. For nondegenerate games, there are an odd number of isolated Nash equilibria. In general, however, the Nash equilibria of a game are not isolated, and instead form closed, connected components, of which there are finitely many (21). That is, *NE* is the disjoint union $NE=\underset{1\leq i\leq n}{⨆}NE_{i}$, where $NE_{i}$ is a component. Recall that a Nash equilibrium is by definition a point from which no player will want to deviate. Hence, for a dynamics φ to be compatible with game *g*, we only require that φ satisfy Nash stationarity: NE(g)⊆Fix(φ), where by Fix(φ), we denote the fixed points of φ, the set {x∈X:φ(t,x)=xfor all t≥0}. That is, the dynamics is compatible with a game if it does not move away from any of the Nash equilibria of the game. Namely, game dynamics encode the strategically stability of all Nash equilibria, i.e., that self-interested agents would not deviate from them. This is a very natural definition, and game dynamics have been studied extensively under this assumption, or variants; see for instance, refs. 21–27; these studies often result in a characterization of Nash components in terms of fixed-point index theory.

Now, given such a dynamical system, what is an appropriate notion of convergence? This is a deep question within topological dynamical systems theory, and a classical tool used to characterize convergence is that of Lyapunov functions. In fact, Lyapunov function arguments are the archetypal strategy for proving convergence to Nash equilibria in games, cf. refs. 28–34. Furthermore, the topological theory of dynamical systems implies that any dynamical system has an appropriate Lyapunov function, as the existence of a complete Lyapunov function is guaranteed by Conley’s fundamental theorem of dynamical systems (15): This theorem states that any dynamical system can be decomposed into the so-called chain recurrent set—the set to which the dynamics converges and on which the Lyapunov function is constant—and a gradient-like part, upon which a Lyapunov function strictly decreases along trajectories; see refs. 15 and 35. This leads us to our definition:

Definition 1: Given game dynamics φ for a game *g*, we say that φ is Nash convergent if there exists a continuous function V:X→[0,1] such that

1. if x∉NE and t>0 then V(φ(t,x))<V(x),
2. if $x,y\in NE_{i}$ for some i, then V(x)=V(y).

**Fig. 1.**
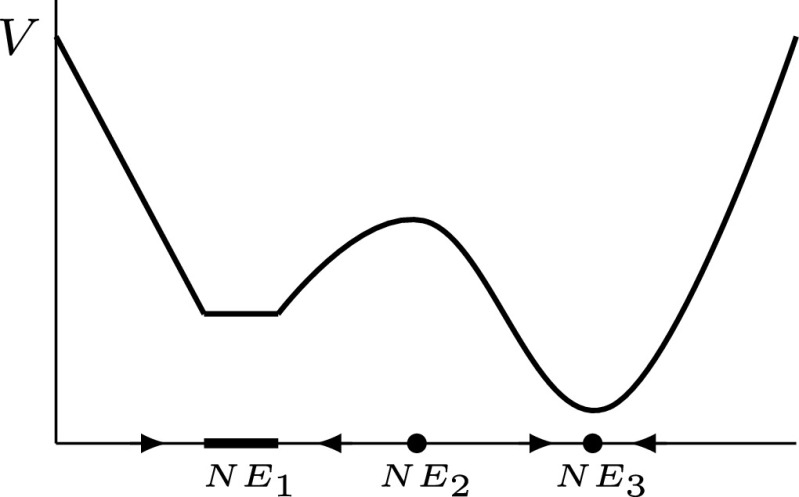
Rendering of Lyapunov function V for Nash convergent game dynamics φ. For game dynamics, the components of Nash equilibria are invariant sets. The function V is constant on the components while points which are not Nash equilibria lie on trajectories between components.

See Fig. 1 for an illustration. That is to say, V is a continuous function on X which is strictly decreasing on trajectories outside *NE* and constant on trajectories within any Nash component; cf. ref. 15.

Now that we know what convergence means, the statement of our main result becomes clearer: There is a game *g* which does not admit any Nash convergent game dynamics. The techniques we use to prove this result come from Conley index theory (15) and require minimal technical assumptions on the space and dynamics. These techniques have not been previously applied to the setting of game dynamics, and provide a powerful framework for analysing the global dynamics of games, the implications of which will be explored in future work. Moreover, up to a basic knowledge of algebraic topology, our paper is self-contained, as all the necessary results are provided in the Notes.

## Dynamical Systems Theory

To prove our result, we make use of the relationship between Lyapunov functions, attractors, and Conley indices. To this end, we introduce all these mathematical concepts and their connections to the extent that they are needed in the proof. We will consider a compact metric space X (for game dynamics, X is the product of the players’ strategy simplices) and let $T^{+}=\left\{t\in T:t\geq 0\right\}$ where T is either R or Z. A dynamical system is a continuous map $\varphi :T^{+}\times X\to X$, such that i) φ(0,x)=x for all x∈X and ii) $\varphi \left(t+t^{\prime },x\right)=\varphi \left(t^{\prime },\varphi \left(t,x\right)\right)$ for all $t,t^{\prime }\in T^{+}$ and x∈X. When T=R, i.e., for continuous-time, φ is called a semiflow; if T=Z, i.e., discrete time, φ is often called a map or discrete time dynamical system.

A basic notion in dynamical systems is that of an attractor, which intuitively is a set to which trajectories converge asymptotically. For a subset Y⊂X, the asymptotic behavior of Y under the dynamics φ is captured by the omega-limit set, formally defined as $\omega \left(Y\right)=\underset{t>0}{⋂}\;\text{cl}(\varphi \left(\left[t,\infty \right),Y\right))$, where cl denotes topological closure. A set A⊂X is an attractor if there exists a neighborhood *U* of A such that ω(U)=A. An attracting block *U* is a closed neighborhood of A such that ω(U)=A and φ(t,U)⊂intU for all t>0. Attracting blocks always exist (36). Finally, the maximal attractor is ω(X).

### Conley Index Theory for Attractors.

Conley index theory deals with the more general concept of an isolated invariant set; however, for our purposes, we need only introduce the Conley index in the limited setting of attractors. The Conley index takes a different form depending upon whether the time is discrete or continuous, and we introduce each as well as prove, separately, both corresponding theorems. We defer the formal exposition of Conley index theory to the Notes, providing for now an intuition for the continuous-time case.

Let $\varphi :T^{+}\times X\to X$ be a semiflow, A be an attractor for φ, and *U* an attracting block of A. The Conley index of A is defined as:

$$CH_{∙}\left(A\right):=H_{∙}\left(U\right).$$

Here, $H_{∙}$ denotes singular homology with integer coefficients. As shown in Proposition 1 of the Notes, the Conley index is independent of the particular choice of *U* and, thus, provides an algebraic topological invariant for A. As X is an attracting block for the maximal attractor, ω(X), its Conley index is defined as:

$$CH_{∙}\left(\omega \left(X\right)\right):=H_{∙}\left(X\right).$$

**Fig. 2.**
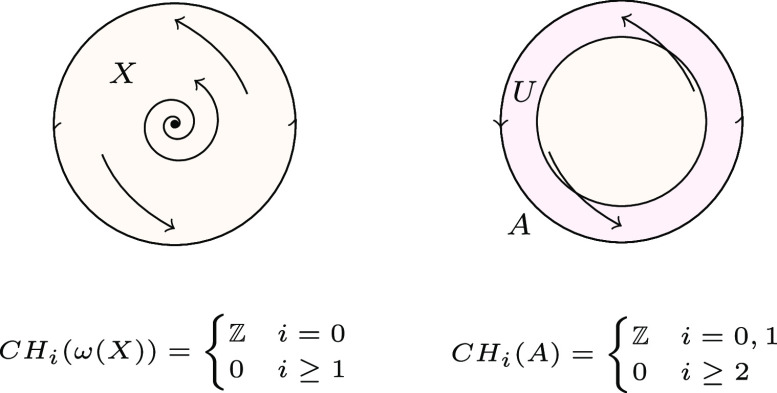
A disk X with semiflow φ, attractor A (stable periodic orbit) and an unstable fixed point in the interior (*Left*). Here, ω(X)=X, and the Conley index of ω(X) is the homology of a point. Attracting block *U* for A which is homotopy equivalent to a circle (*Right*). The Conley index of A is the homology of a circle.

Fig. 2 provides elementary Conley index computations for a semiflow.

## Impossibility in Game Dynamics

In this section, we prove our main result, the impossibility theorem, splitting the proof into the cases of continuous time (Theorem 1) and discrete time (Theorem 2).

Theorem 1. *There exists a game g that does not admit continuous-time Nash convergent game dynamics.*

***Proof***: Define *g* to be the following bimatrix game as in (21, 25):

[1] $$\begin{array}{c} \left(R,C\right)=\left(\begin{array}{ccc} 1,1 & 0,-1 & -1,1\\ -1,0 & 0,0 & -1,0\\ 1,-1 & 0,-1 & -2,-2 \end{array}\right). \end{array}$$

By way of contradiction, suppose that there exist game dynamics φ which are Nash convergent. For *g* given in Eq. **1**, the set of Nash equilibria forms a topological circle, i.e., $S^{1}$ (21). Thus, there is a single component of Nash equilibria, which is homeomorphic to $S^{1}$. As φ is Nash convergent, there exists a Lyapunov function V:X→[0,1] such that for any x∉NE and any t>0, V(x)>V((φ(t,x))>0. Without loss of generality, we may assume that $V^{-1}\left(0\right)=NE$. For any λ∈(0,1], setting $U_{\lambda }=V^{-1}(\left[0,\lambda \right])$, we have that $NE=\omega (U_{\lambda })$. Thus, *NE* is an attractor for φ. In fact, as $X=V^{-1}\left(\left[0,1\right]\right)$, and NE=ω(X), *NE* is the maximal attractor. It follows from the continuity of V that $U_{\lambda }$ is a closed attracting block for *NE* for any λ∈(0,1]. In particular, we can first calculate the Conley index of *NE* as:

[2] $$\begin{array}{c} CH_{i}\left(NE\right)=H_{i}\left(X\right)=\left\{\begin{array}{cc} Z & i=0\\ 0 & i>0. \end{array}\right. \end{array}$$

**Fig. 3.**
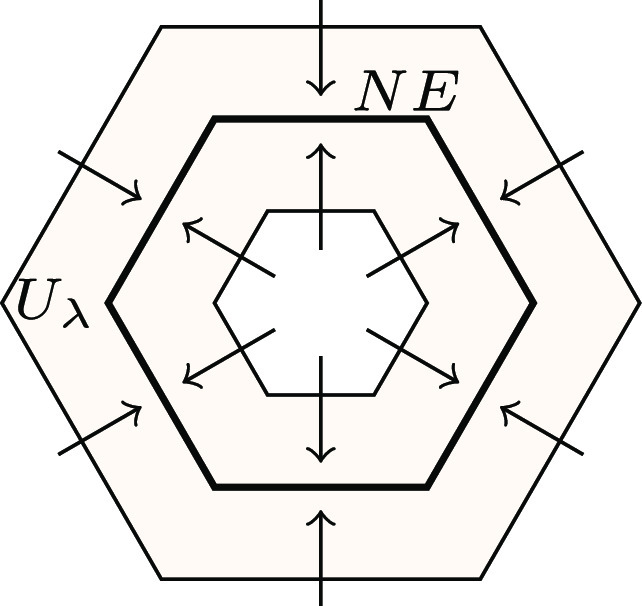
Rendering of circle of Nash equilibria (*NE* lives in four dimensions); $NE=V^{-1}\left(0\right)$. Attracting block $U_{\lambda }$ for attractor *NE* and Lyapunov function V ensures that the dynamics converges to *NE*.

Now, the inclusion $\iota :NE↪U_{\lambda }$ induces the homomorphism on homology $\iota_{∙}:H_{∙}\left(NE\right)\to H_{∙}\left(U_{\lambda }\right)$. Intuitively, we can choose λ small enough so that $U_{\lambda }$ is a tight enough neighborhood of *NE* so that the circle of Nash equilibria which generates the 1-dimensional hole in $H_{1}\left(NE\right)$ still exists as a 1-dimensional hole in $U_{\lambda }$, as in Fig. 3. More formally, if w is the generator of $H_{1}\left(NE\right)=Z$, for λ>0, sufficiently small $U_{\lambda }$ is a small neighborhood of *NE* and the image $\iota_{1}\left(w\right)$ cannot be the boundary of any 2-chain in $U_{\lambda }$. Thus, the map $\iota_{1}:H_{1}\left(NE\right)\to H_{1}\left(U_{\lambda }\right)$ is injective. As $U_{\lambda }$ is an attracting block, we can compute the Conley index of *NE* as:

[3] $$\begin{array}{c} CH_{∙}\left(NE\right)=H_{∙}\left(U_{\lambda }\right). \end{array}$$

However, as the map $\iota_{1}$ is injective, we have that

[4] $$\begin{array}{c} CH_{1}\left(NE\right)=H_{1}\left(U_{\lambda }\right)\neq 0. \end{array}$$

However, by Proposition 1 the Conley index^*^ is an invariant of *NE* and does not depend on the choice of attracting block. Therefore, this is a contradiction and such a φ cannot exist.

We now consider the discrete time case, which is proved in the same fashion, using the Conley index for maps, the background for which is provided in the Notes.

Theorem 2. *There exists a game g that does not admit discrete time Nash convergent game dynamics.*

***Proof***: Consider again the game of Eq. **1**. Assuming that there are Nash convergent discrete time game dynamics for *g*, there exists a Lyapunov function V:X→[0,1], and without loss of generality, we may assume $V^{-1}\left(0\right)=NE$. Once again, it follows that *NE* is an attractor, and in fact the maximal attractor of φ and for any λ∈(0,1], $U_{\lambda }=V^{-1}(\left[0,\lambda \right])$ is an attracting block for *NE*. We will reach the desired contradiction by comparison of the Conley indices computed via X and $U_{\lambda }$ for sufficiently small λ. First, as X is an attracting block for *NE*, we can use it to compute the Conley index for *NE*. Note that $H_{∙}\left(X\right)$ is computed in Eq. **2** and induced map $f_{X}:H_{∙}\left(X\right)\to H_{∙}\left(X\right)$ is the identity map (taking the generator of the connected component to itself). The Conley index of *NE* is given by $\left(L_{∙}\left(X\right),\chi_{X}\right)$, which is:

$$L_{∙}\left(X\right)=M_{∙}\left(X\right)=H_{∙}\left(X\right),$$

and the automorphism $\chi_{X}$ is the identity map. In particular, $L_{1}\left(X\right)=H_{1}\left(X\right)=0$. Again, the inclusion $\iota :NE↪U_{\lambda }$ induces a map on homology $\iota_{∙}:H_{∙}\left(NE\right)\to H_{∙}\left(U_{\lambda }\right)$ and when λ is sufficiently small, the map $\iota_{1}:H_{1}\left(NE\right)\to H_{1}\left(U_{\lambda }\right)$ is injective. Thus, $H_{1}\left(U_{\lambda }\right)\neq 0$. By Nash stationarity, *NE* is a circle of fixed points, and thus, the map f(x):=φ(1,x) induces the identity map ${f|}_{\mathrm{NE}}:H_{∙}\left(NE\right)\to H_{∙}\left(NE\right)$. In particular, there is a commutative diagram:




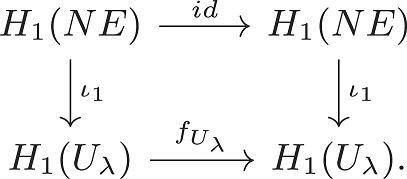




Letting w be the generator of $H_{1}\left(NE\right)=Z$, and $w^{\prime }=\iota_{1}\left(w\right)$, the diagram implies $f_{U_{\lambda }}\left(w^{\prime }\right)=w^{\prime }$. It is elementary that this implies that $L_{1}\left(U_{\lambda }\right)\neq 0$. However, Proposition 2 implies that the Conley index is independent of attracting block. Thus, this is a contradiction, and there cannot exist such a φ.

Remark 1: An inspiration for our results is the work of Benaïm et al. (25), wherein they make the statement, without complete proof, that there are games *g* and such that for all φ within a class of multivalued dynamical systems, it cannot be the case that NE(g)=CR(φ), where CR(φ) is the chain recurrent set (see ref. 15) of φ. The argument sketched in ref. 25 ultimately rests on the development of a fixed point index for components of Nash equilibria and its comparison to the Euler characteristic of the component. However, the argument is incomplete (indeed, the reader is directed to two papers, one of which is a preprint that seems to have not been published). Instead, for our result, we assume Nash convergence, which is in fact a weaker condition as the chain recurrent set has such a Lyapunov function obeying (1) and (2), and thus, if CR(φ)=NE, φ is Nash convergent. Thus, as a corollary of Theorem 1, there exists a game which does not admit game dynamics for which the chain recurrent set coincides with the set of Nash equilibria.

Remark 2: In discussions with Gil Kalai and, independently, Venkat Anantharam, the following dynamics came up: In the two-dimensional disk $D=\{x,y:x^{2}+y^{2}\leq 1\}$, consider the continuous dynamics, described by the vector field $f\left(x,y\right)=\left(0,1-x^{2}-y^{2}\right)$. Obviously, the only fixed points are the boundary of the disk, and there are no cycles. Now, homeomorphically map this to a topological disk T whose boundary is the hexagonal Nash component of *g* and move every other point of the domain toward T, smoothening appropriately the transition to the motion of T. Is this not then a dynamical system that converges to the Nash equilibria of *g*, contradicting our main result?

The topological theory of dynamical systems provides a negative answer: There can be no Lyapunov function supporting this dynamics, and the chain recurrent set of the dynamics is all of T, instead of its boundary. Indeed, a similar example is contained in ref. 15.

## Nondegenerate Games and Approximate Equilibria

### Nondegenerate Games.

Our impossibility result in the previous section is constructed around a degenerate normal-form game with a continuum of equilibria. What if the game is nondegenerate?

Theorem 3. *For any nondegenerate game g, there is dynamical system φ_g_ satisfying Nash stationarity which is Nash-convergent.*

***Proof***: Since *g* is nondegenerate, it has an odd number of isolated Nash equilibria (37). Thus, in this case, the Nash equilibria themselves are the maximal components. Fix one such equilibrium and call it y; e.g., $y=NE_{0}$. We next define $\varphi_{g}$ in terms of y. We shall define it at point x implicitly, in terms of the direction of motion, and the speed of motion; if this is done, $\varphi_{g}\left(t,x\right)$ is easily computed through integration on t. The direction of motion is the unit vector of y−x: The dynamics heads to y. The speed of motion is defined to be $c\cdot D_{g}\left(x\right)$, where c>0 is a constant, and by $D_{g}\left(x\right)$, we denote the deficit at x: the sum, over all players, of the difference between the best-response utility at x, and the actual utility at x. It is clear that $D_{g}\left(x\right)\geq 0$, and it becomes zero precisely at any Nash equilibrium; thus, $\varphi_{g}$ has the property of Nash stationarity. Define $V\left(x\right)=||x-{y||}_{2}$ as the Lyapunov function. It is clear that V strictly decreases along trajectories off of *NE* (such trajectories head to y). Therefore, φ is Nash convergent.

Remark 3: It’s worth remarking on why $\varphi_{g}$ as constructed above is not Nash-convergent for the degenerate game in Eq. **1**. In this case, there are points x∉NE that belong on orbits converging to the chosen Nash equilibrium y but come from a Nash equilibrium z≠y. However, the Lyapunov function V must have V(z)=V(y) as both z and y belong to the same component. Therefore, V could not be strictly decreasing along such trajectories. In fact, these dynamics are similar to the example dynamics discussed in the previous remark.

Now note that the algorithm for specifying $\varphi_{g}$ requires finding y, a PPAD-complete (and FIXP-complete for more than two players) problem. We believe that the dependence is inherent:

Conjecture 1. *The computational task of finding, from the description of a game g, either a degeneracy of g or an algorithm producing the direction of motion and speed of a dynamical system satisfying Nash stationarity and Nash convergence is PPAD-hard (and FIXP-hard for three or more players).*

We believe this is an important open question in the boundary between computational complexity, game theory, and the topology of dynamical systems, whose resolution is likely to require the development of complexity-theoretic techniques pertinent to dynamical systems. We further suspect that, if the sought dynamics are constrained to be utility-increasing, i.e., that given stationary opponents the agents move in the direction of increasing utility whenever possible, the difficulty of finding such a Nash convergent dynamics is increased to NP-hard.

### Approximate Nash Equilibria.

Next, it may seem plausible that the difficulty of designing Nash convergent game dynamics can be overcome when only ϵ-approximation is sought, for some ϵ>0. Recall that an ϵ-Nash equilibrium is a mixed strategy profile in which all players’ utilities are within an additive ϵ>0 of their respective best response. We show that our impossibility theorem extends to this case as well. Let us denote by $NE_{\epsilon }\left(g\right)$ the set of ϵ-approximate Nash equilibria of a game *g*; there are a finite number of components of ϵ-approximate Nash, so that once again, $NE_{\epsilon }\left(g\right)=\underset{1\leq i\leq n}{⨆}NE_{\epsilon ,i}$. We say that a dynamical system φ has the property of ϵ-Nash stationarity with respect to a game *g* if $NE_{\epsilon }\left(g\right)\subset \;\text{Fix}\left(\varphi \right)$, and that φ is ϵ-Nash convergent if there is a Lyapunov function V that is constant on these components, and strictly decreasing along trajectories if $x\notin NE_{\epsilon }$. We begin with a lemma.

Lemma 1. *Consider the bimatrix game* g=(R,C) *and* ϵ>0. *For any* $\epsilon^{\prime }>0$ *and game* $g^{\prime }=\left(R^{\prime },C^{\prime }\right)$ *such that* $||R-R^{\prime }{\left|\right|}_{F}\leq \epsilon^{\prime }$ *and* $||C-C^{\prime }{\left|\right|}_{F}\leq \epsilon^{\prime }$ *(where* $\left|\right|\cdot {\left|\right|}_{F}$ *denotes the Frobenius norm), we have* $NE_{\epsilon }\left(g\right)\subset NE_{2\epsilon^{\prime }+\epsilon }\left(g^{\prime }\right)$.

***Proof***: The argument for the first player follows, as for any $\left(x,y\right)\in NE_{\epsilon }\left(g\right)$ any i:

$$\begin{array}{cc} e_{i}^{T}R^{\prime }y^{T}-x^{T}R^{\prime }y & ={\left(e_{i}-x\right)}^{T}\left(R^{\prime }-R\right)y+e_{i}^{T}Ry-x^{T}Ry\\ & \leq \left|\right|e_{i}-{x||}_{2}\left|\right|R^{\prime }-{R||}_{F}{\left||y|\right|}_{2}+\epsilon \leq 2\epsilon^{\prime }+\epsilon . \end{array}$$

The inequality for the second player is proved similarly.

Theorem 4. *There is a game g and an ϵ > 0 so that g does not admit any dynamics that are both ϵ-Nash stationary and ϵ-Nash convergent. In fact, the set of such games has positive measure.*

***Proof***: Consider again the Kohlberg–Mertens game Eq. **1**. Define the function

$$\begin{array}{c} h_{g}\left(z,y\right)=\max_{i}\;\max\left(e_{i}^{T}Ry-z^{T}Ry,z^{T}Ce_{i}-z^{T}Cy\right). \end{array}$$

**Fig. 4.**
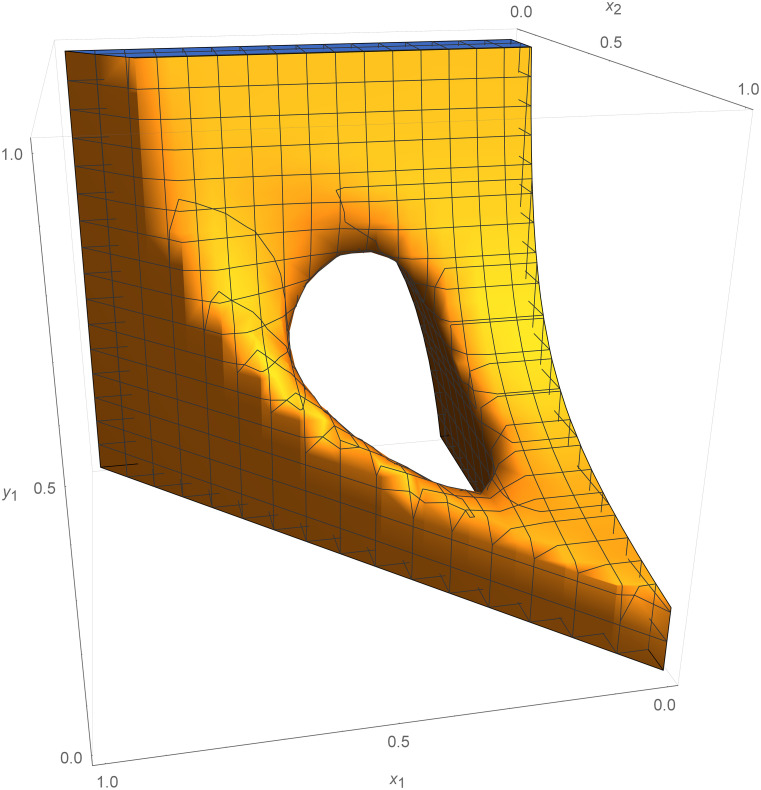
Three-dimensional projection of $NE_{\epsilon }\left(g\right)$ when *g* is the Kohlberg–Mertens game Eq. **1**, and for utility-normalized ϵ=0.09. The object depicted is homotopy equivalent to $S^{1}$ (a circle).

Then, $h_{g}$ is a continuous function such that for any ϵ>0, we have that $NE_{\epsilon }\left(g\right)=h_{g}^{-1}(\left[0,\epsilon \right])$; in particular, $NE\left(g\right)=h^{-1}\left(0\right)$. For a sufficiently small ϵ>0, the inclusion $NE\left(g\right)\subset NE_{\epsilon }\left(g\right)$ induces an injection on homology $\iota_{1}:H_{1}\left(NE\left(g\right)\right)\to H_{1}\left(NE_{\epsilon }\left(g\right)\right)$. Assuming that there exists ϵ-Nash convergent ϵ-Nash stationary dynamics φ, the arguments in the proofs of Theorem 1 and Theorem 2 can be repeated to show that $NE_{\epsilon }\left(g\right)$ is a maximal attractor for φ, and that the Conley index for $NE_{\epsilon }\left(g\right)$ computed via X and a small neighborhood of $NE_{\epsilon }\left(g\right)$ do not agree, which is a contradiction. See Fig. 4 for a projection of $NE_{\epsilon }\left(g\right)$.

We now show that the set of such games which admit no $\frac{\epsilon }{2}$-Nash convergent $\frac{\epsilon }{2}$-game dynamics has positive measure. Consider perturbations of the game *g* where each utility value of the normal form of *g* is perturbed independently, so that there is an 18-dimensional vector of perturbations whose 2-norm is no more than $\frac{\epsilon }{4}$. It is clear that this set of games has positive measure, and for any such perturbed game $g^{\prime }=\left(R^{\prime },C^{\prime }\right)$, we have $||R-R^{\prime }{\left|\right|}_{F}\leq \frac{\epsilon }{4}$ and $||C-C^{\prime }{\left|\right|}_{F}\leq \frac{\epsilon }{4}$. We can apply Lemma 1 twice to get the containment $NE\left(g\right)\subset NE_{\frac{\epsilon }{2}}\left(g^{\prime }\right)\subset NE_{\epsilon }\left(g\right)$. Consider the associated induced maps on homology

$$H_{∙}\left(NE\left(g\right)\right)\overset{\iota_{1}}{\to }H_{∙}\left(NE_{\frac{\epsilon }{2}}\left(g^{\prime }\right)\right)\overset{\iota_{2}}{\to }H_{∙}\left(NE_{\epsilon }\left(g\right)\right).$$

The map $H_{∙}(\iota_{2}){}^{\circ}H_{∙}(\iota_{1})=H_{∙}(\iota_{2}{}^{\circ}\iota_{1})$ is an injection, which implies that $H(\iota_{1})$ is injective and therefore $H_{∙}\left(NE_{\frac{\epsilon }{2}}\left(g^{\prime }\right)\right)\neq H_{∙}\left(X\right)$. Therefore, a similar argument as above shows that if $NE_{\frac{\epsilon }{2}}\left(g^{\prime }\right)$ is a maximal attractor, the Conley indices computed via a small neighborhood and by X do not agree, which completes the proof.

## Conclusion

We have argued that the notion of the Nash equilibrium is fundamentally incomplete for describing the global dynamics of games. More precisely, we have shown that there are games in which no dynamics can be Nash convergent, and thus, the concept of the Nash equilibrium cannot completely account for the long-term dynamical behavior in games. Moreover, this is true even when one relaxes the focus from Nash equilibria to approximate Nash equilibria.

Ultimately, in view of the present results about the limitations of the Nash equilibrium, the meaning of the game, as well as the meaning of general economic systems, should be sought elsewhere, perhaps with a closer focus on agent dynamics as echoed in ref. 38. Indeed, detailed studies of several learning dynamics in ref. 10 suggest that the structure of best-reply paths and cycles holds important clues for making progress in this direction. Specifically, the existence of best-reply cycles predicts nonconvergence of six different learning dynamics supported by experiments with human subjects. See also refs. 39 and 40 for proposed general solution concepts in game theory, alternatives to the Nash equilibrium, that are inspired by game dynamics and the topological theory of dynamical systems. Moving beyond game theory, a natural and exciting direction for future work is the pursuit of such dynamics-inspired solution concepts more generally in the social and even the natural sciences.

Regarding a more robust understanding of game dynamics, Conley theory provides a natural choice. For instance, as player utilities are not derived from first principles, but are presumably rough approximations of reality, it is natural that the appropriate mathematical objects for the analysis of game dynamics should be robust to perturbation—in the words of Conley (15), “*...if such rough equations are to be of use it is necessary to study them in rough terms.*” This suggests the use of further ideas from dynamical systems theory, for instance, the concept of Morse decomposition, which allows for a coarse, multiscale description of global dynamics. Morse decompositions are partially ordered sets of isolated invariant sets which possess a duality theory with lattices of attractors and in addition have an associated homological theory using the Conley index (15). We believe that these ideas could provide a robust theory of the global dynamics of games.

## Notes: Conley Index Theory

The classical reference for Conley index theory is Conley’s original monograph (15). A standard reference for homotopy theory and singular homology theory is ref. 41.

### Continuous Time.

Let $\varphi :T^{+}\times X\to X$ be a semiflow, A an attractor for φ, and *U* an attracting block for A. The Conley index of the attractor A is defined as follows:

$$CH_{∙}\left(A\right):=H_{∙}\left(U\right),$$

where $H_{∙}$ denotes singular homology with integer coefficients. Most importantly for this paper, the Conley index is an invariant of A and is independent of the particular choice of *U* (15, 42). An elementary proof of this is as follows.

Proposition 1. *If* U *and* $U^{\prime }$ *are attracting blocks for attractor* A*,* *then* U *and* $U^{\prime }$ *are homotopy equivalent. In particular,* $H_{∙}\left(U\right)≅H_{∙}\left(U^{\prime }\right)$*,* *and thus, the Conley index is well defined.*

***Proof**:* It is elementary that if U and $U^{\prime }$ are attracting blocks, $U\cap U^{\prime }$ is an attracting block; see also ref. 36. Thus, we may assume without loss of generality that $U\subset U^{\prime }$. Now, since *U* and $U^{\prime }$ are attracting blocks for the same attractor A, there exists some T>0 such that $\varphi (T,U^{\prime })\subset U$. It is straightforward to check that the pair of maps $\varphi \left(T,\cdot \right):U^{\prime }\to U$ and inclusion $\iota :U\to U^{\prime }$ form a homotopy equivalence between *U* and $U^{\prime }$, as for any $t^{*}>0$, the map $\varphi (t^{*},\cdot )$ is homotopic to the identity map via the homotopy $\left(x,s\right)\mapsto \varphi \left(s\cdot t^{*},x\right)$.

### Discrete Time.

In the discrete time case, the Conley index takes a slightly more complex form, as the dynamical system itself no longer provides a homotopy equivalence. Let $\varphi :T^{+}\times X\to X$ be a discrete time dynamical system, A be an attractor for φ, and *U* be an attracting block for A. Define f:X→X via f(x):=φ(1,x), then f(U)⊂U, and, with slight abuse of notation, there is an induced map $f_{U}:H_{∙}\left(U\right)\to H_{∙}\left(U\right)$, i.e., the well-defined homomorphism given by $f_{U}\left(\left[x\right]\right)=\left[f\left(x\right)\right]$. The construction, introduced in ref. 43, proceeds in two stages and depends on the notions of generalized kernel and generalized image. Define the generalized kernel of $f_{U}$ as:

$$\;\text{gker}\left(f_{U}\right):=\underset{n>0}{⋃}\ker f_{U}^{n},\;\;\text{and}\;M_{∙}\left(U\right):=H_{∙}\left(U\right)/\;\text{gker}\left(f_{U}\right).$$

The map $f_{U}$ induces an injective map $f_{U}^{\prime }:M_{∙}\left(U\right)\to M_{∙}\left(U\right)$. Now consider the generalized image of $f_{U}^{\prime }$:

$$L_{∙}\left(U\right):=\underset{n>0}{⋂}f_{U}^{\prime }(M_{∙}\left(U\right)).$$

The map $f_{U}^{\prime }$ induces a surjection on the generalized image: $\chi_{U}:L_{∙}\left(U\right)\to L_{∙}\left(U\right)$. Moreover, as the restriction of $f_{U}^{\prime }$, $\chi_{U}$ is an injection. Thus, $\chi_{U}$ is an automorphism. The Conley index of *U* is defined as the pair:

$$\begin{array}{c} \;\text{Con}\left(A\right):=(L_{∙}\left(U\right),\chi_{U}), \end{array}$$

and is independent of choice of attracting block *U*.

Proposition 2. *Let* φ *be a discrete dynamical system,* A *be an attractor for* φ *and* U *and* $U^{\prime }$ *attracting blocks for* A*. The Conley index is independent of choice of* U*. In other words, there exists an isomorphism* $\psi :L_{∙}\left(U\right)\to L_{∙}\left(U^{\prime }\right)$ *so that the following diagram commutes:*

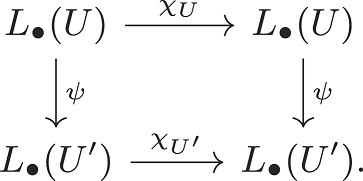




***Proof***: We may again assume $U\subset U^{\prime }$ without loss of generality. Moreover, since *U* and $U^{\prime }$ are attracting blocks for A, there is some n>0 we have $f^{n}\left(U^{\prime }\right)\subset U$. It is straightforward that the inclusion $U\subset U^{\prime }$ induces a map ι:$L_{∙}\left(U\right)\to L_{∙}\left(U^{\prime }\right)$ and the map $f^{n}$ induces a map s:$L_{∙}\left(U^{\prime }\right)\to L_{∙}\left(U\right)$ so the following diagram commutes:




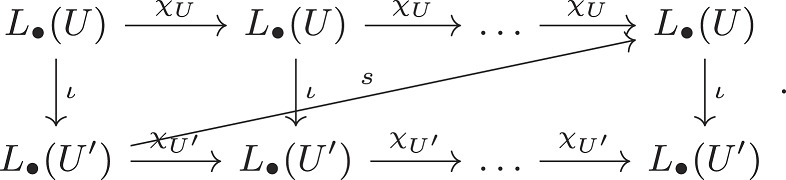




Now, $\chi_{U}$ and $\chi_{U}^{\prime }$ are automorphisms, and as $\chi_{U}^{n}=f^{n}\;{}^{\circ}\;\iota$, we have that ι is a injection, and further as $\chi_{U^{\prime }}^{n}=\iota \;\;{}^{\circ}f^{n}$, ι is a surjection. Thus, ι is an isomorphism, and the Conley index is independent of choice of attracting block.

## Data Availability

There are no data underlying this work.

## Footnotes

^*^For dynamics with memory, the impossibility result follows from the inequality between the Conley index of their statespace (e.g., two copies of a product of simplices in ref. 19) and their respective target set (e.g., two copies of the same Nash equilibrium).
